# Memory for musical tones: the impact of tonality and the creation of false memories

**DOI:** 10.3389/fpsyg.2014.00582

**Published:** 2014-06-12

**Authors:** Dominique T. Vuvan, Olivia M. Podolak, Mark A. Schmuckler

**Affiliations:** ^1^Department of Psychology, International Laboratory for Brain, Music, and Sound Research, Université de MontréalMontreal, QC, Canada; ^2^Department of Psychology, University of Toronto ScarboroughToronto, ON, Canada

**Keywords:** tonality, music perception, melody, false memory, schema, availability, distinctiveness

## Abstract

Although the relation between tonality and musical memory has been fairly well-studied, less is known regarding the contribution of tonal-schematic expectancies to this relation. Three experiments investigated the influence of tonal expectancies on memory for single tones in a tonal melodic context. In the first experiment, listener responses indicated superior recognition of both expected and unexpected targets in a major tonal context than for moderately expected targets. Importantly, and in support of previous work on false memories, listener responses also revealed a higher false alarm rate for expected than unexpected targets. These results indicate roles for tonal schematic congruency as well as distinctiveness in memory for melodic tones. The second experiment utilized minor melodies, which weakened tonal expectancies since the minor tonality can be represented in three forms simultaneously. Finally, tonal expectancies were abolished entirely in the third experiment through the use of atonal melodies. Accordingly, the expectancy-based results observed in the first experiment were disrupted in the second experiment, and disappeared in the third experiment. These results are discussed in light of schema theory, musical expectancy, and classic memory work on the availability and distinctiveness heuristics.

## Introduction

The study of expectancy, simply defined as “the anticipation of upcoming information based on past and current information” (Schmuckler, 1997) has a long history in cognitive psychology. For instance, Mowrer (1938) discovered that he could evoke anxiety in animals using the presentation of aversive stimuli, and introduced the term “preparatory set” to describe the state of anxious expectancy evoked in his animal subjects as a result of these stimuli. Later, Neisser (1976), studying human perception, was inspired by Piaget (Piaget and Walsh, 1971) and Bartlett (1932) to develop a theory of action-oriented perception, central to which was the idea of the anticipatory schema. Neisser defined anticipatory schemata as mental structures that prepared individuals for action as well as readying them for certain kinds of sensory input. From this perspective, schemata drive expectancies, and in some ways, “schema” and “expectancy” can be considered two approaches to describing the same cognitive process, with that process using the schematic structure to produce an expectation for an event. Expectancy has been studied in numerous contexts, including perceptual processing (e.g., Brown and Hildum, 1956; Dykes and Pascal, 1981), attention (e.g., Posner, 1980; Downing, 1988), linguistic processing (e.g., Fodor et al., 1974; Mills, 1980; McClelland and O'Regan, 1981; Anderson and Pearson, 1984), and the cognition of narratives (e.g., Bartlett, 1932; Mandler and Johnson, 1977; Bransford, 1979).

One area within which expectancy has been a particular focus of research for the last two decades is music cognition. In fact, music is in many ways an ideal medium with which to study expectancy. Music, especially Western tonal-harmonic music, is based on a well-defined structure that has been thoroughly studied and described by theorists (e.g., Schenker, 1954; Laitz, 2008). This music theoretic work is important to the scientific study of expectancy in multiple ways. First, theoretical descriptions of musical structure (or schemata) and function can inform our investigations into the psychological processing of expectancy. For example, the theoretical notion that pitch is organized into different hierarchical levels has been productively studied in psychological contexts (see Krumhansl, 1990 for a review). Second, musical structure allows for a large range of complexity. Musical pieces can vary in both pitch and in time. Both these dimensions can be manipulated in terms of texture, from simple (e.g., melodies or isochronous rhythms) to complex (chord progressions or intricate meters), and combined in myriad interactions. Music thus provides ideal material for strictly controlled internally valid experimentation as well as more generalizable, externally valid study. Finally, music, like all auditory stimuli, unfolds over time. Thus, the ability to predict what comes next in a musical piece is fundamental to its perception. For these reasons, the study of expectancy in music has been a fruitful avenue of research that encapsulates various theoretical and methodological approaches.

Work on musical expectancy has been plentiful for a number of years, on both a theoretical and experimental basis. Theoretical approaches to musical expectancy have taken a variety of forms (Meyer, 1956; Lerdahl and Jackendoff, 1983; Narmour, 1989, 1990, 1992; Huron, 2006). By and large this work has been devoted to understanding the formation of musical expectancy, from a music theoretic point of view, along with the impact of expectancy formation on listeners' subsequent identification of structural relations, musical meaning, or musical emotion.

Empirical work has been similarly prolific over this time period (Carlsen et al., 1970; Carlsen, 1981, 1982; Jones, 1981, 1982; Bharucha and Stoeckig, 1986, 1987; Boltz, 1989, 1993; Schmuckler, 1989, 1990; Jones, 1990; Bharucha, 1994; Krumhansl, 1995; Schellenberg, 1996, 1997; Tekman and Bharucha, 1998; Tillmann et al., 1998, 2000, 2003; Larson, 2002, 2004; Bigand et al., 2003; Margulis, 2005; Ockelford, 2006; Pearce and Wiggins, 2006, 2012; Marmel et al., 2008, 2010; Marmel and Tillmann, 2009; Thorpe et al., 2011; Ockelford and Sargeant, 2012). This work has also explored multiple aspects of musical expectancy, and has similarly highlighted the factors underlying expectancy formation, along with the impact of expectancy formation for on-going musical processing and subsequent musical memory. Schmuckler, for instance, identified the operation of high-level, structural factors in driving expectations, as well as the operation of relatively low-level, bottom-up perceptual processes (Schmuckler, 1989, 1990). In terms of the former influence, the principal factor involved the role played by the musical tonality (described subsequently) of the excerpt in question whereas the latter influence centered around the operation of basic Gestalt-like pattern processes (Meyer, 1956; Narmour, 1989, 1990, 1992).

Briefly described, tonality refers to the system of relations between musical elements (e.g., musical tones, chords, and keys) in the Western musical system. These elements are organized into a hierarchy structured around a central reference pitch, such that every pitch class (numbered 0–11, these tones comprise the complete set of notes used in Western music) has a well-defined level of theoretical and perceived psychological stability with respect to this reference pitch (Schmuckler, 2004, 2009). Within Western music two forms of tonality are typically used—“major” tonality and “minor” tonality. Although these two different forms are both organized hierarchically, they differ in terms of which tones fall at which levels of the hierarchy. Table 1 presents the tonal hierarchy structure for both a major and a minor tonality.

**Table 1:** **Tonal hierarchies for major and minor keys**.

**Tonal hierarchy level**	**Pitch class**
**MAJOR**	
Tonic	0
Tonic triad members	4 7
Diatonic tones	2 5 9 11
Non-diatonic tones	1 3 6 8 10
**MINOR (HARMONIC)**	
Tonic	0
Tonic triad members	3 7
Diatonic tones	2 5 8 11
Non-diatonic tones	1 4 6 9 10

Classic work by Krumhansl et al. (Krumhansl, 1979, 1990, 2000; Krumhansl and Shepard, 1979; Krumhansl and Kessler, 1982; Cuddy and Badertscher, 1987; Halpern et al., 1996) has demonstrated the psychological existence of this hierarchy. For instance, Krumhansl and Kessler (1982) presented listeners with a tonality defining context, followed by a single “probe” tone, and asked listeners to judge how well the probe belonged with the preceding context. These authors found that listeners' belongingness judgments mirrored the theoretical hierarchy of tonal stability as commonly assumed by music theorists. Accordingly, this work confirms that listeners have robust mental representations of tonality that guide their perception of music, and as such, also influence the nature of the expectancies formed when listening to music.

Tonality has been found to influence expectancy processes involving both the on-line processing of musical information, as well as subsequent memory for musical materials. In terms of the former research focus, the most thoroughly investigated aspect has to do with expectancy priming effects. In the original exploration of this topic, Bharucha and Stoeckig (1986, 1987) presented listeners with a prime chord followed shortly thereafter by a target chord, and asked them to make some judgment regarding this target chord (i.e., whether the chord was in-tune vs. out-of-tune, or in major vs. minor form). The primary manipulation employed in these studies involved the tonal relation between the prime and target; in some cases the prime and target were closely related tonally whereas in other cases the prime and target were tonally unrelated. These authors observed substantial priming effects, with targets that were related to the primes processed more quickly and accurately than targets that were unrelated to their primes. This tonal priming effect has been both replicated and expanded upon by Bharucha et al. (Bharucha and Stoeckig, 1986, 1987; Tekman and Bharucha, 1998; Tillmann et al., 1998, 2000, 2003; Bigand et al., 2003; Marmel et al., 2008, 2010; Marmel and Tillmann, 2009). Generally, this research has demonstrated that when listening to complex musical passages, listeners develop expectations about what is to come next based on the tonality of the music, with these expectations significantly influencing the speed and accuracy with which listeners process such information.

In terms of the latter research focus, some scholars have directly linked memory to expectancy, arguing that the abiding purpose of memory is actually to generate predictions based on past experience (e.g., Hawkins and Blakeslee, 2007; Jones and Pashler, 2007). Somewhat surprisingly, there are only a handful of studies examining the relation between tonality, expectancy, and musical memory, although there has been a great deal of work examining the impact of tonality on memory more generally (Dowling, 1978; Cuddy et al., 1979, 1981; Bartlett and Dowling, 1980; Cuddy and Lyons, 1981; Dowling and Bartlett, 1981; DeWitt and Crowder, 1986; Dowling et al., 1995; Halpern et al., 1995, 1998; Schulze et al., 2012; Albouy et al., 2013; see Halpern and Bartlett, 2010, for a review). Overall, this work has demonstrated that tonal structure, relative to atonal structure (i.e., an absence of tonality), produces better memory for musical materials. Cuddy and Lyons (1981), for instance, found that memory for a standard melody was best for melodies that were highly tonal, compared with melodies that were of a more ambiguous tonality. Interestingly, effects of tonality on musical memory have also been found for amusics (Albouy et al., 2013), although the impact in this case has been more in terms of response time to answer in the memory task, as opposed to the memory task itself. Regardless, all of this work demonstrates a recurring relation between memory for melodies and musical tonality.

Other work has looked at the impact of tonality on memory for individual tones. For instance, Krumhansl (1979) investigated the effect of tonality on memory for single pitches by presenting listeners with a standard tone, followed by an intervening sequence of pitches, and then a final comparison tone. The primary manipulation in this study involved the intervening sequence, with these pitches either conforming or not conforming to a musical tonality. Krumhansl found that tones that ranked highly within the tonality were better remembered than tones that ranked lowly within the tonality, particularly when the intervening sequence was tonal.

As already discussed, there have not been many investigations directly examining the impact of expectancy formation on musical memory, although there are at least two experimental projects that have explored this issue. Schmuckler (1997) asked listeners to provide expectancy ratings to a range of melodic endings, and found that melodies with expected endings were better remembered than those with unexpected endings. Interestingly, this work found no effect of tonality on memory for the melodies, but the stimuli of this study were all tonal and thus variation in tonality between melodies were restricted in range. More recently, Curtis and Bharucha (2009) investigated this question within the context of examining how the processing of musical excerpts from one's own culture differs from that of an unfamiliar culture (i.e., exercising a different musical structure and organization). These authors employed a recognition memory paradigm originally used by Deese (1959) and more recently reintroduced by Roediger and McDermott (1995), in which Western-enculturated listeners heard a series of tones drawn either from the Western tonal system, or the Indian Bhairav scale, followed by a test tone, and were asked to make a speeded judgment about whether or not that test tone was included in the earlier sequence. Listeners were more likely to falsely remember context-congruent than context-incongruent tones in the Western stimuli, with this pattern reversed for the Bhairav stimuli. Because an incongruent note in the Bhairav context was a congruent one in the Western context, this reversal demonstrates that listeners interpreted the tone series derived from both familiar and unfamiliar pitch structures through the lens of the Western tonal schema. Accordingly, this work implicates the role of expectancy generation on listeners' memory.

Interestingly, the approach adopted by Curtis and Bharucha (2009) provides some important methodological advantages over the technique used by Schmuckler (1997). First, simplifying the structure of the target event to a single tone, as opposed to a set of tones (e.g., a melodic ending) allows improved control over the nature of this target, and thus more exact assessment of the nature of listeners' expectancies on musical memory. Moreover, this technique enables a more direct assessment of the relation of the target to the context material (e.g., how often does the target or cues to the target occur in the context, if at all?). An important consequence of this increased control is that multiple levels of expectancy (rather than the dichotomous expected vs. unexpected) can be tested, thus providing a finer-grained understanding of the relation between expectancy and memory in music. Given these advantages, the current studies adopted the methodology of Curtis and Bharucha (2009) to explore the relation between expectancy formation and subsequent musical memory.

What are the possible ways in which expectancy, and more specifically expectancies based on musical tonality, might influence subsequent musical memory? In considering the possible relations between these two domains, Schmuckler (1997) proposed two different means by which tonal expectancies might influence memory. The first, or “congruency” account, is based on schema theory (Neisser, 1976; Bharucha, 1994) which posits that one is better prepared to process events that are congruous with one's schema, as opposed to events that are incongruous with the schema. An early form of this idea was developed in Bartlett's (1932) classic naturalistic studies of memory using the native American folk story, “The War of the Ghosts.” During retelling, participants tended to distort the details of the story, such that the reproduction was made more similar to a story schema with which participants were familiar, with details that were congruent with this schema added, and details that were incongruent omitted or transformed to be more congruent. Thus, memory was not a faithful storing of past events, but rather a reconstructive process dependent upon schematic expectancies based on previous experience.

Further evidence of the reconstruction inherent in memory comes from the extensive literature on false memory (see Brainerd and Reyna, 2005 for a review). The field of false memory research comprises a broad range of subject areas and disparate methodological approaches, from clinical psychiatry, to behavioral psychology, to cognitive neuroscience. However, a common thread across these streams of research is the concept of schematic processing influencing the creation of false memories. For instance, Deese (1959) discovered that given a list of words to memorize, participants often recalled words that were not on the list. Importantly, the likelihood of any word being falsely recalled depended on its schematic association to words that had appeared on the list. Picking up on this thread, Roediger and McDermott (1995) showed that false memories could be deliberately induced by asking participants to study word lists created according to a particular theme. During the test phase, participants demonstrate higher false alarms for lures that corresponded to that theme than those that do not. These false memories are resistant to explicit warnings and immediate testing (McDermott and Roediger, 1998), and have been attributed to associative processing, by which activation spreads among related concepts (Roediger et al., 2001a). Turning to the field of eyewitness memory, Loftus' pioneering work has found that post-experience suggestion can cause participants to misremember events if they are congruent with the gist of their memory, or are compatible with participants' preconceptions regarding the people or place involved (Loftus and Pickrell, 1995; Belli and Loftus, 1996; Loftus, 1996, 2003, 2005). Interestingly, these results are consistent with the reported effects of social contagion on false memory (Roediger et al., 2001b).

Given such reconstructive processes, the congruency account predicts that tones that are expected would be remembered well, whereas tones that are unexpected would be remembered poorly. Accordingly, tones at the higher levels of the tonal hierarchy (such as appear at the top levels in Table 1), which are more expected in tonal melodies, would be better remembered than tones at lower levels of the hierarchy, which are less expected (Schmuckler, 1989). The congruency account also makes an interesting subsidiary prediction. Specifically, stemming from work on false memory, this account predicts that highly expected (i.e., tones at a higher level of the tonal hierarchy) would be falsely remembered as having occurred in melodies even when they were not, in fact, sounded. In general the predictions of the congruency account are consistent with previous work on memory for melodies (Krumhansl, 1979; Schmuckler, 1997), as well as Tversky and Kahneman's (1973) classic work on the availability heuristic in which people tend to judge events that are more easily brought to mind as more likely to occur.

The availability heuristic has been found to operate across a wide range of situations, including risk assessment (Folkes, 1988; Agans and Shaffer, 1994; Keller et al., 2006; Sunstein, 2006), education (Billings and Schaalman, 1980; Fox, 2006), ethical decision making (Hayibor and Wasieleski, 2009), financial decision making (Kliger and Kudryavtsev, 2010), judgments of the self and others (Cervone, 1989; Schwarz et al., 1991; Rothman and Hardin, 1997), mental imagery (Carroll, 1978), and subliminal priming (Gabrielcik and Fazio, 1984). Thus it would not be surprising to observe availability effects in musical memory. In this case, highly expected tones are more available, and thus more easily brought to mind, which would make them better remembered when they did occur, and falsely remembered when they do not.

As an alternative to a congruency account, it is also possible that memory will be influenced by the distinctiveness of a given target item. This “distinctiveness” account emanates from the classic von Restorff effect (von Restorff, 1933; Hunt, 1995), wherein isolating an item from its background enhances memory for that item later on. With respect to musical processing, this explanation posits that tones that are highly unexpected (i.e., tonally unstable tones) within a context would be better remembered because they “pop out” of the surrounding context and are thus better attended and encoded. Theoretically, this account is related to Schacter et al.' work (Schacter et al., 1999, 2001; Dodson and Schacter, 2001, 2002; Schacter and Wiseman, 2006) showing that the processing of distinctive features of an event can improve memory for that event later on; this phenomenon has been labeled the distinctiveness heuristic. Moreover, the voluminous body of work investigating the importance of perceptual pop out across a wide array of domains and stimulus dimensions provides further support for the importance of distinctiveness as driving attention, and thus subsequent memory. This work has been predominantly visual (e.g., Treisman and Gelade, 1980; Prinzmetal, 1981; Treisman, 1982; Treisman and Schmidt, 1982; Prinzmetal et al., 1986; Enns, 1990; Nothdurft, 1991; Maljkovic and Nakayama, 1994; Wang et al., 1994; Li, 1999; Quinlan, 2003), but pop out phenomena have also been documented in the auditory (Woods et al., 1994, 2001; Cusack and Carlyon, 2000; Zimmer et al., 2000; Janata et al., 2003; Dyson and Alain, 2004; Magne et al., 2005; Van der Burg et al., 2008), and haptic (Plaisier et al., 2008) domains.

Interestingly, work on distinctiveness processing typically considers distinctiveness as synonymous with perceptual salience. However, von Restorff theorized that any form of distinctiveness should lead to enhanced memory (Hunt, 1995). Hence, this theory could also lend itself to distinctiveness based on musical tonality. It is worth noting that tonal distinctiveness differs from feature-based perceptual salience in that it is a higher-order attribute. That is, rather than relying on a single dimension, tonality relies on the interaction of pitch with time, in terms of note durations, and also on the relations between different pitches. Again, this idea does converge with work investigating higher-order pop out effects in visual search, such as has been found with affective distinctiveness. For example, Hansen and Hansen (1988) reported that angry faces popped out from a crowd, although the veracity of this effect is a matter of controversy (Hampton et al., 1989; Purcell et al., 1996; Fox et al., 2000; Hershler and Hochstein, 2005, 2006; VanRullen, 2006). In the auditory domain, very little is known about attentional pop out effects. Thus, these experiments contribute new knowledge regarding pop out in auditory contexts, as well as higher-order pop out in general.

The current set of studies investigated whether tonal expectancies would influence memory for individual components (i.e., tones) of a musical context. Additionally, if tonality did in fact have an impact on memory, these studies attempted to disentangle which of the two previously discussed approaches—the congruency or distinctiveness account—would better predict listeners' memory. It is important to note, however, that although these accounts make different predictions, they are not necessarily mutually exclusive. That is, it is possible that both congruency and distinctiveness could simultaneously influence memory for tones. Along these lines, one might see better memory for both highly expected (tonally stable) and highly unexpected (tonally unstable) tones, relative to tones of intermediate expectancy (tones lying at intermediate levels of the tonal hierarchy). As an aside, the idea that expectancy can be subdivided into varying degrees of expectation does have precedents both in terms of its psychological existence (see Schmuckler, 1989, for examples of melodic and harmonic expectancy), and in terms of its impact on musical processing and responses (Schmuckler and Boltz, 1994).

The current studies tested these ideas employing a modified version of the method of Curtis and Bharucha (2009) in which listeners heard a melody followed by a target tone, and were asked whether or not the target tone had occurred in the preceding melody (the actual occurrence of the target within the melody varied across trials). Target tones were chosen such that they were of high expectation, medium expectation, or low expectation; these varying levels correspond to tones of high tonal stability, medium tonal stability, and low tonal stability (see Schmuckler, 2004, 2009). Looking across the three experiments in this series, the relative strength of the underlying tonality was manipulated by employing melodies that induced a strong, perceptually stable tonality (Experiment 1), melodies that induced a tonality that was of weaker perceptual stability (Experiment 2), or melodies that induced no perceived tonality at all (Experiment 3).

## Experiment 1: note memory in major tonality melodies

The goal of the first experiment was to determine whether, in fact, expectancies generated by a tonal melody would influence memory for single tones. Toward this end, this study employed a major tonal context in order to generate a strong representation of tonality (e.g., Dowling, 1978, 1991; Krumhansl and Shepard, 1979; Krumhansl, 1979, 1990; Krumhansl and Kessler, 1982; Bharucha and Stoeckig, 1986, 1987; Schmuckler, 1989, 1997; Bharucha, 1994; Marmel et al., 2008). Memory for three different target tones was investigated, with these tones chosen based on their representing varying levels of perceived psychological and tonal stability with a major tonality (see Table 1). Specifically, these tones represented highly expected, moderately expected, and unexpected tones in a major key context. Based on the previously discussed theoretical approaches, these differing levels of expectancy should produce varying patterns of performance for both memory rates and false alarm rates.

### Methods

#### Participants

Twenty participants (14 females; mean age = 18.7 years, *SD* = 0.33 years) were recruited from the University of Toronto Scarborough community using the introductory psychology participant pool, and compensated for their participation with course credit. The following descriptive statistics were calculated for all 20 participants. These participants had an average of 3.0 years of formal musical training (*SD* = 0.5 years), with four participants reporting no training. Participants had an average of 0.2 years of musical theory training (*SD* = 0.1 years), with 16 participants reporting no exposure to music theory. With respect to other musical activity, on average, participants listened to music for 13.3 h per week (*SD* = 2.9 h), and played music for 1 h per week (*SD* = 0.5 h). None of the participants had ever participated in a music psychology experiment before, nor did any participants report a familiarity with the music cognition research literature. Finally, none of the participants reported having absolute pitch.

#### Stimuli

All melody and probe tone stimuli were produced using a grand piano sound in MakeMusic Inc. (2009). All melodies were composed in the key of G major, and were based on melodies taken from two American folk song collections (Jackson, 1964; Ohrlin, 1973). The melodies were presented in only a single key, based on previous research that has shown that the tonal representations generalize across tonal centers (Krumhansl and Kessler, 1982). Moreover, the use of G major in all melodies ensured that the tonal center, and thus tonal expectancy, was strongly established during each block. To ensure that the melodies did indeed induce a predominant tonality of G major key, all melodies were analyzed using the Krumhansl-Schmuckler key-finding algorithm (Krumhansl, 1990; Schmuckler and Tomovski, 2005). The algorithm indicated that G major was the highest correlated key for all melodies, with a mean correlation with G major across all melodies of *r*_(22)_ = 0.80 (*SD* = 0.08). The second highest key correlation for each melody was most often E minor, then D major, and then G minor, which are all highly tonally related to G major (see Krumhansl, 1990). However, given that the G major correlation was significantly higher than the second highest key correlation across melodies, *t*_(71)_ = 16.53, *p* < 0.00000001, we can be confident that the melodies strongly elicited the perception of G major.

Each melody was four bars long with four beats per bar (4/4 time signature), and was in total between 14 and 16 beats long (the number of beats occupied by notes in the fourth bar varied from 2 to 4). Melodies were played at a tempo of 120 beats per min (i.e., a quarter tone = 500 ms), resulting in melodies of between 7 and 8 s in length. All melodies ranged in pitch from B3 (246.94 Hz) to B5 (987.77 Hz), and ended on the tonic tone (G).

Two factors were manipulated across these melodies. The first factor was *Target Presence*, with the target either present in or absent from the melody. If the target was present it only appeared once in the melody. Second was the factor of *Target Note*. Present targets could consist of a high expectancy note (in G major this was the note D, or pitch class 7; see Table 1), a moderate expectancy note (E, or pitch class 9), and a low expectancy note (D#/Eb, or pitch class 8). Across the set of melodies the target note could occur in one of four positions: measure 2, beat 2; measure 2, beat 3; measure 3, beat 2; or measure 3, beat 3. Varying the temporal position of the target was important in preventing listeners from simply anticipating when during the melody the possible target note might occur, and thus directing heightened attention solely to that temporal location. If the target was absent, it did not occur at any point in the melody (e.g., Target Absent, Target Note = D means that D never occurred).

**Figure 1:**
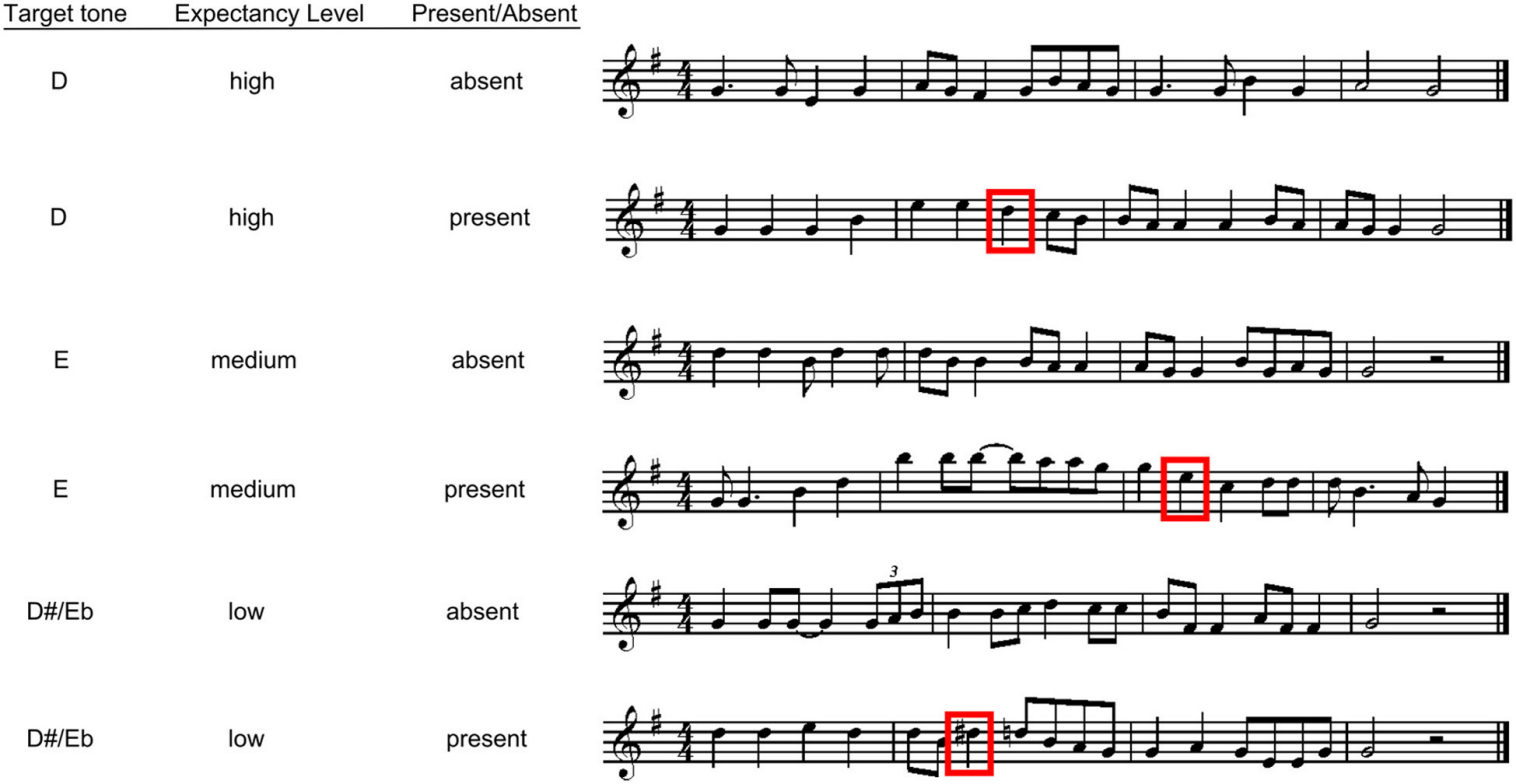
**Examples of experimental stimuli for Experiment 1**. The boxes indicate present targets.

The combination of target notes varying in their expectancy (i.e., high, moderate, low expectancy) and the four temporal positions produced 12 possible configurations for melodies containing the target note. Three melodies were created for each configuration, giving rise to 36 melodies in all with the target present (12 for each expectancy level). Thirty-six new melodies were then composed which did not include the targets (to balance the number of target absent with target present melodies). Thus, there were 72 melody stimuli in all; examples of these melodies can be seen in Figure 1, and all melodies (with contour and interval information for all targets) are available in Supplementary material.

Following each melody listeners heard a one second probe tone. This probe was identical in pitch to the target note in the target present melodies. For the target absent melodies the pitch of the probe tone was one of the three possible target tones, and was transposed to the range of the melodic context.

#### Apparatus

Stimuli were presented to participants using an Intel Pentium 4 personal computer, with code written and run in MATLAB 7.0 (Moler, 2004), using the Cogent toolbox (Romaya, 2002). The visual components of the experiment were viewed on an LG Flatron L1710S monitor, and the auditory components were heard through Sennheiser HD 280 pro headphones connected to a Creative Sound Blaster Audigy 2 ZS soundcard. Participants were given the opportunity to adjust the volume of the auditory stimuli to a comfortable listening level. Responses were collected using the “1” (for present) and “0” (for absent) keys on the computer keyboard.

#### Procedure

Participants were told that they would hear a melody, followed shortly thereafter by a single probe tone. They were told to listen carefully to the melody and probe, and to then indicate whether or not they had heard the probe tone in the previously presented melody. Participants heard three blocks of 72 trials, with the order of these trials randomized within each block. Thus, altogether listeners heard 216 experimental trials. Prior to beginning the experimental trials, listeners received five practice trials (randomly chosen from the 72 experimental trials) and had the opportunity to ask the experimenter questions regarding the task. Following the experimental trials, listeners completed a survey regarding their musical experience. The entire experimental session lasted approximately 1 h.

### Results and discussion

Participant responses (“target present” vs. “target absent”) were used to calculate the hit rate (correctly detecting the presence of the target when it was present) and false alarm rate (incorrectly indicating the presence of the target when it was absent) for each target condition. These hit and false alarms rates were in turn used to calculate the bias-free sensitivity index *d*', and the bias index c according to signal detection theory (MacMillan and Creelman, 2005). *d*' reveals the separation between the means of the signal (“target present”) and noise (“target absent”) distributions, and thus indicates how well participants were able to discriminate between trials in which the target had occurred in the melody and trials in which it had not. Therefore, *d*' can be treated here as a proxy for memory performance, with larger values of *d*' corresponding to better memory. c indicates the participant criterion for answering “target present” vs. “target absent,” with *c* = 0 indicating no bias, *c* < 0 indicating a liberal bias (more likely to answer “present”), and *c* > 0 indicating a conservative bias (more likely to answer “absent”).

In order to control for the effects of musicianship, all ANOVA analyses reported for Experiment 1 were performed with musical training (in years) as a covariate. There was never a significant main effect of musical training, nor were any interactions with musical training significant, all *p* > 0.05.

**Figure 2:**
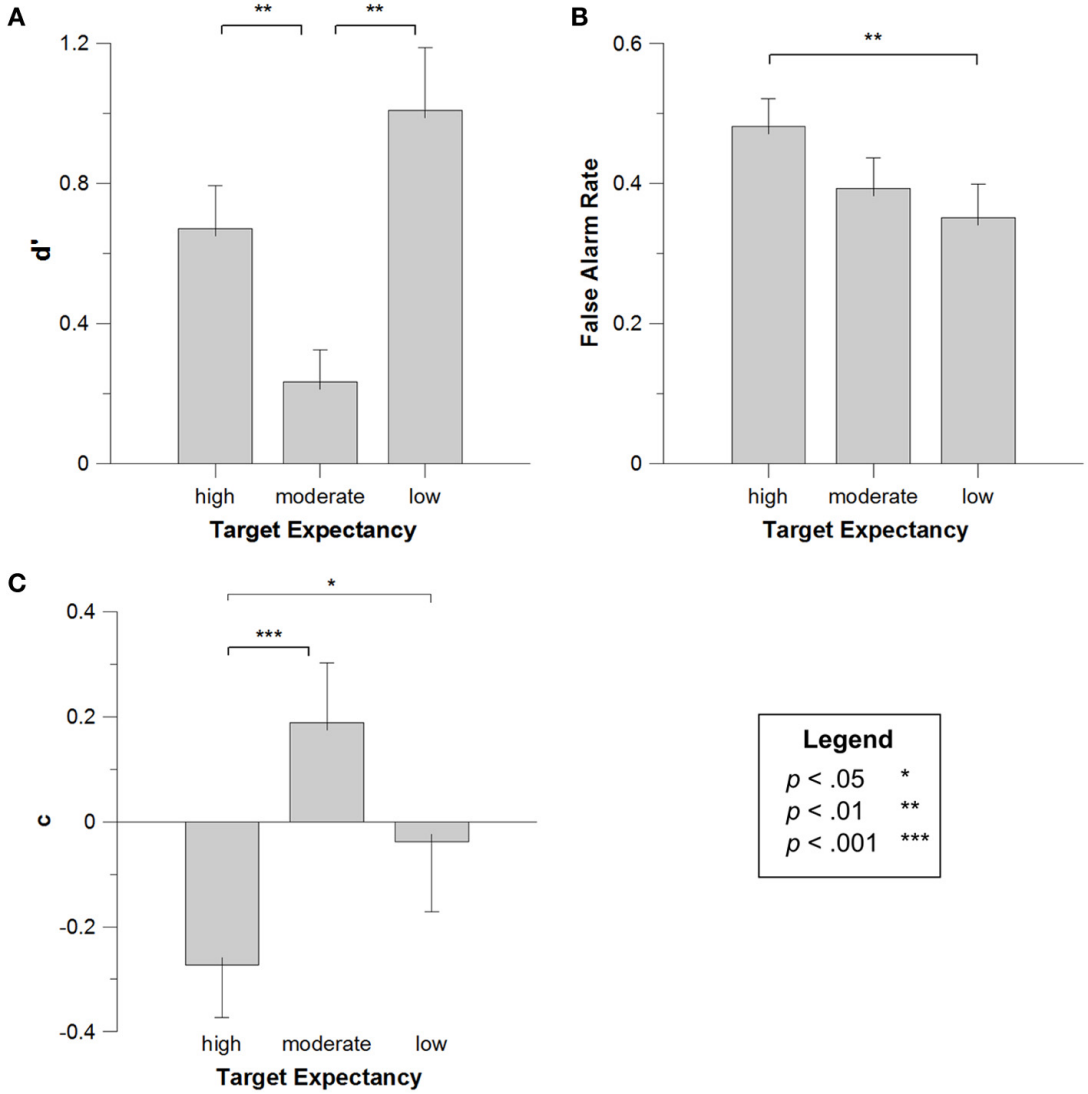
**d' **(A)**, false alarm rate **(B)** and c **(C)** for high, moderate, and low expectancy targets in Experiment 1**. Error bars depict the standard error of the mean.

First, we confirmed that participant performance, as measured by *d*', was significantly better than chance (*d*' = 0), *t*_(19)_ = 6.613, *p* < 0.001. Next, *d*' data for each participant in each target condition were submitted to a One-Way repeated measures ANOVA, with the within-subjects factor of *Target Note* (high vs. medium vs. low expectancy). The effect of target was significant, *F*_(2, 38)_ = 11.041, *MSE* = 0.267, *p* < 0.001, *η*^2^_*p*_ = 0.380. Figure 2A presents the means (and *SE*s) for the *d*'s as a function of target note. Multiple Bonferroni-corrected comparisons (critical *p* = 0.05/3 = 0.017) showed that this effect was due to both high expectancy targets and low expectancy targets being better-remembered than moderate expectancy targets, *t*_(19)_ = 3.194, *p* = 0.005; *t*_(19)_ = 3.922, *p* = 0.001. Listeners' memory for high and low expectancy targets was not significantly different, *t*_(19)_ = 2.013, *p* = 0.058, though there was a trend toward slightly better memory for targets that were unexpected than those that were expected. These results provide evidence for both the congruency and distinctiveness accounts, with expectancy affecting memory for tones by privileging processing of items that are schema-congruent as well as items that are schema-incongruent^1^. To our knowledge, no previous studies have demonstrated that both congruency and distinctiveness can be simultaneously operative in memory; therefore, this constitutes a novel finding in the field of memory.

In order to test the hypothesis that highly expected targets would be falsely remembered, false alarm rates for each participant in each target condition were submitted to a One-Way repeated measures ANOVA, again employing the factor of *Target Note*. This factor was marginally significant, *F*_(2, 38)_ = 2.314, *MSE* = 0.021, *p* = 0.113, *η*^2^_*p*_ = 0.114; Figure 2B presents the means (and *SE*s) for this effect. Given our predictions regarding false alarms, we conducted multiple Bonferroni-corrected comparisons (critical *p* = 0.05/3 = 0.017) which confirmed that highly-expected targets producing more false alarms than unexpected targets, *t*_(19)_ = 3.428, *p* = 0.003, and marginally more false alarms than moderately-expected targets, *t*_(19)_ = 2.016, *p* = 0.058. This result confirms an important prediction of the congruency account—that tonal schemata encourage listeners to reconstruct what they heard with schema-congruent tones, leading to an elevated false alarm rate for the highly expected target. These data are consistent with past work in false memory (Brainerd and Reyna, 2005), and represent one of the first reports of false memory effects in a musical context (see also Curtis and Bharucha, 2009), and, notably, the only report specifically assessing highly-learned, acculturated music.

Finally, *c* values for each participant in each target condition were submitted to a One-Way repeated measures ANOVA with *Target Note* as a factor. There was no overall effect of *Target Note, F*_(2, 38)_ = 1.789, *MSE* = 0.160, *p* = 0.182, *η*^2^_*p*_ = 0.090. However, planned comparisons (critical *p* = 0.017) indicated that participants had a marginally more liberal response criterion for trials with high expectancy tones than medium expectancy tones, *t*_(19)_ = 2.164, *p* = 0.043, and a significantly more liberal response for trials with high expectancy tones than low expectancy tones, *t*_(19)_ = 4.300, *p* < 0.001. Figure 2C presents the means (and *SE*s) for this effect. This result is consistent with the results from the false alarms analysis, with more liberal bias leading to higher false alarms for high expectancy targets.

Overall, these findings support the idea that listeners' expectancies for melodies, in this case, formed on the basis of perceiving a strong tonality, will influence subsequent memory for the components (i.e., the individual tones) of the melodies. What is intriguing is that the impact of tonally-driven expectancy formation was multi-faceted, with listeners demonstrating better memory for tones that are strongly consistent with the perceived tonality (i.e., a congruency effect) and presumably driving attention to tones that are strongly inconsistent with the perceived tonality (i.e., a distinctiveness account). For both effects, however, the critical aspect of processing leading to these memory effects involves the formation of a robust representation of tonality by which expectancies can be generated. Recognition of this central component leads naturally to the question of what would happen to expectancy effects on memory if the tonal representation was not so robust. Experiments 2 and 3 address this question.

## Experiment 2: note memory in minor tonality melodies

Assuming that the memory differences observed in Experiment 1 were indeed the result of expectancies generated by the perceived tonality of the melodies, then if listeners heard sequences that were less robust in producing tonal expectancies the memory differences would be correspondingly influenced. One straightforward method of manipulating the strength of listeners' tonal representations is to employ melodic contexts in a minor tonality rather than a major one. Previous work has shown that listeners' cognitive representations of the minor tonality is weaker than that of the major tonality (Krumhansl et al., 1982; Harris, 1985; Delzell et al., 1999; Vuvan and Schmuckler, 2011), possibly because three different versions of the minor tonal structure can be represented simultaneously (Vuvan et al., 2011). As an example, Vuvan and Schmuckler (2011) found that listeners were able to generate highly accurate images of a major tonality based on a cue tone. In contrast, listeners' auditory images of a minor tonality were dramatically less robust, indicating that such contexts are significantly less psychologically stable. Within the current paradigm, employing minor melodic contexts should serve to decrease the fidelity of the tonal schema, thereby weakening the effects of tonality-based expectancies on memory performance.

### Methods

#### Participants

Twenty participants (16 females; mean age = 20.0 years, *SD* = 5.2 years) who had not participated in Experiment 1 were recruited from the University of Toronto Scarborough community using the introductory psychology participant pool, and compensated for their participation with course credit. The following descriptive statistics were calculated for all 20 participants. These participants had an average of 6.0 years of formal musical training (*SD* = 3.9 years), and an average of 3.5 years of musical theory training (*SD* = 3.2 years). With respect to other musical activity, on average, participants listened to music for 12.3 h per week (*SD* = 13.1 h), and played music for 1.8 h per week (*SD* = 2.6 h). None of the participants had ever participated in a music psychology experiment before, nor did any participants report a familiarity with the music cognition research literature. Finally, none of the participants reported having absolute pitch.

#### Stimuli, apparatus, and procedure

**Figure 3:**
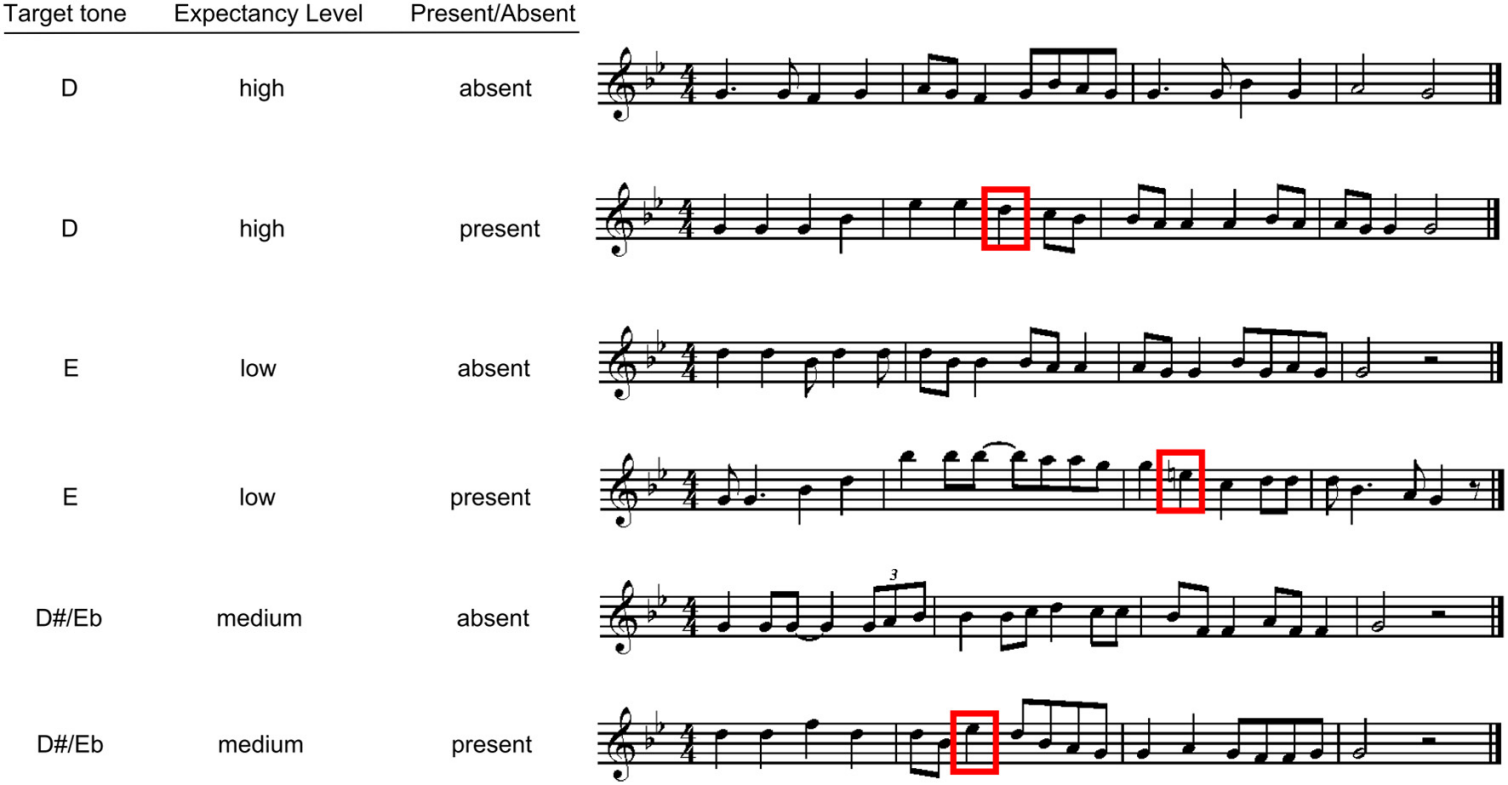
**Examples of experimental stimuli for Experiment 2**. The boxes indicate present targets.

The apparatus and procedure for this experiment were identical to that of Experiment 1. The only difference between the two studies involved the stimuli for this experiment, with these melodies now presenting a minor key context, as opposed to a major key. To create the minor key stimuli, the melodies from Experiment 1 were altered by changing specific tones within each melody to be consistent with a minor tonal hierarchy (see Table 1), without altering the contour. The changed tones were never target tones. Because of the structural differences between major and minor tonalities, the moderate expectancy target (E, or pitch class 9) from Experiment 1 now became the low expectancy target, whereas the low expectancy target (D#/Eb, or pitch class 8) from Experiment 1 now became the moderate expectancy target. The high expectancy target (D, pitch class 7) remained the same across the two experiments (see Table 1). Examples of these melodies can be seen in Figure 3; all melodies are available in Supplementary material.

To ensure that the alterations to these melodies did, in fact, reliably modify the tonality, the Krumhansl-Schmuckler key-finding algorithm (Krumhansl, 1990) was applied to each melody. The algorithm indicated that each melody had a minimum correlation of *r*_(22)_ = 0.65 with G minor, with a mean correlation with G minor for all melodies of 0.82 (*SD* = 0.07). The second highest key correlation for each melody was most often G major, then B flat major, and then E flat major, which are all highly tonally related to G minor (see Krumhansl, 1990). However, given that the G minor correlation was significantly higher than the second highest key correlation across melodies, *t*_(71)_ = 15.771, *p* < 0.00000001, we can be confident that the melodies strongly elicited the perception of G minor.

### Results and discussion

As in Experiment 1, *d*' and *c* values were calculated based on hit (correct detection of the target when present) and false alarm (incorrect indication of the target when it was absent) rates. In order to control for the effects of musicianship, all ANOVA analyses reported for Experiment 2 were performed with musical training (in years) as a covariate, all *p* > 0.05.

**Figure 4:**
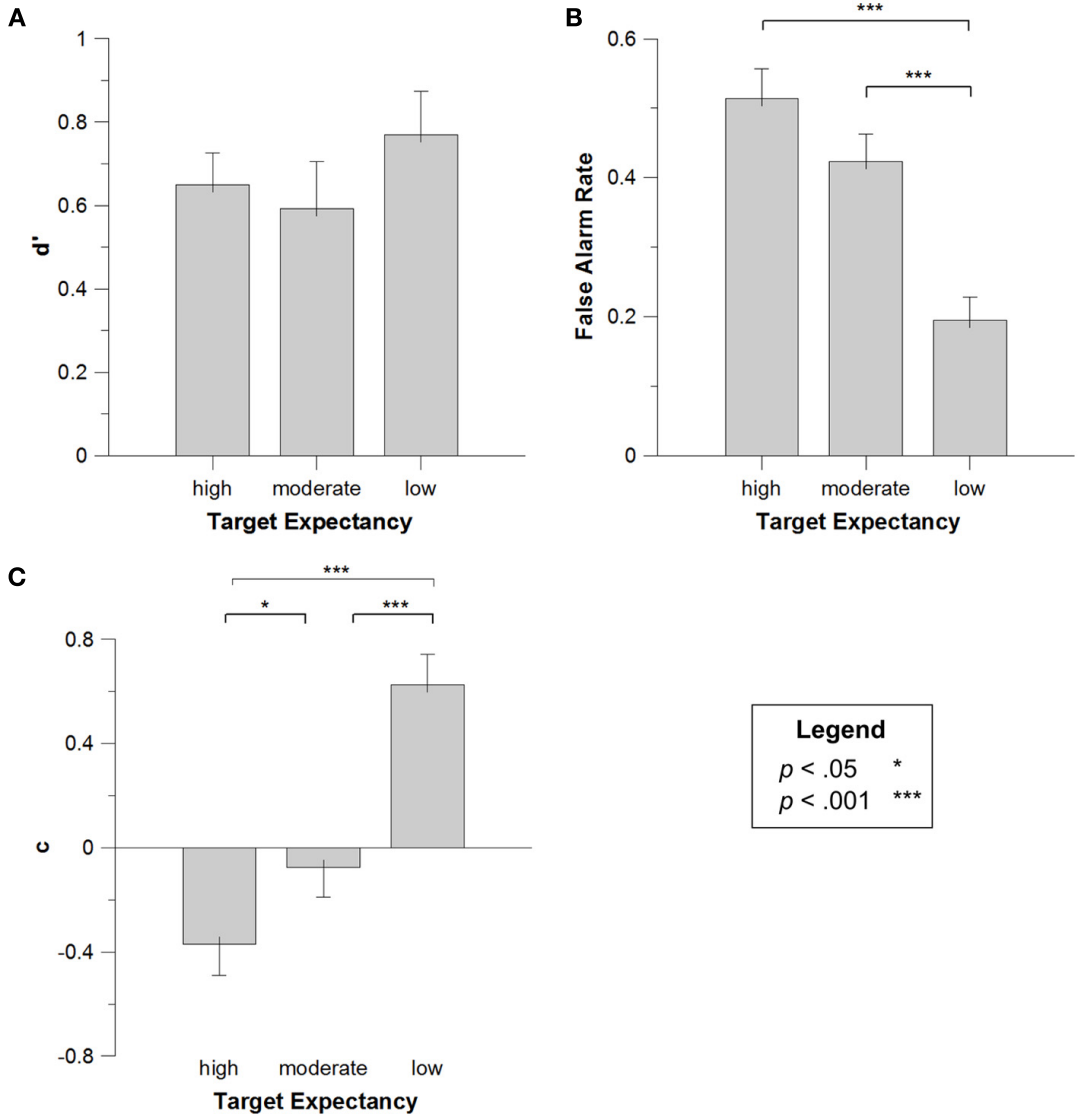
***d'* (A), false alarm rate (B) and *c* (C) for high, moderate, and low expectancy targets in Experiment 2**. Error bars depict the standard error of the mean.

First, we confirmed that participant performance, as measured by *d*', was significantly better than chance (*d*' = 0), *t*_(19)_ = 9.481, *p* < 0.001. Next, *d*' values were submitted to a One-Way repeated measures ANOVA, with the within-subjects factor of *Target Note* (high vs. medium vs. low expectancy). This analysis failed to reveal any effect of the differing targets on *d*'s for minor tonality melodies, *F*_(2, 38)_ = 0.400, *p* = 0.673; Figure 4A presents the means (and SEs) for *d*'s as a function of target tone. Thus, in contrast to Experiment 1, the weaker tonal representations of the minor keys failed to influence memory performance, presumably due to less robust expectancy generation in a minor key (this point will be returned to in the general discussion).

Next, false alarm rates were submitted to a One-Way repeated measures ANOVA with *Target Note* as a factor. In contrast to the d' findings, this analysis did reveal a significant effect of target note, *F*_(2, 38)_ = 4.519, *MSE* = 0.018, *p* = 0.018, *η*^2^_*p*_ = 0.201; Figure 4B presents the means (and *SE*s) for false alarm rates as a function of target tone. Multiple Bonferroni-corrected comparisons (critical *p* = 0.017) revealed that this effect was driven by significantly more false alarms for expected than unexpected targets (see Figure 4B). This was true for both highly expected targets, *t*_(19)_ = 6.122, *p* < 0.001, and moderately expected targets, *t*_(19)_ = 6.948, *p* < 0.001. As an aside, there was a tendency toward more false alarms to high expectancy targets, relative to moderately expected targets, *t*_(19)_ = 2.064, *p* = 0.053, although this difference was not significant after correcting for multiple comparisons. As expected, weakening the tonality of the melodic context by switching from major to minor led to a disruption of the expectancy-based memory effects observed in Experiment 1. Specifically, although differential expectancy for the three targets tones failed to influence *d*', there continued to be an effect of expectancy on false alarm rates for the targets. Admittedly, the pattern in this study was somewhat more complex, with both high and moderate expectancy targets producing increased false alarm rates, relative to low expectancy targets, whereas only high expectancy targets led to increased false alarms in Experiment 1.

One speculative explanation for this finding is that the strong major tonal context in Experiment 1 induced a more differentiated expectancy gradient, with the high expectancy event in the current study clearly distinguishable in its perceived expectancy than the moderate and low expectancy note. In contrast, the minor tonality might have produced a more generalized distinction between diatonic and non-diatonic tones, but not as strong a differentiation between tones at the top levels of the hierarchy (i.e., between high and moderate expectancy events). In this regard it is intriguing to note that the classic tonal hierarchy ratings of Krumhansl and Kessler (1982) actually show a decrease in perceived goodness of fit for pitch class 7 (the high expectancy tone in this study) within a minor key, relative to its perceived fit in a major context. Such a pattern would be consistent with less differentiation between this pitch class and the moderate expectancy pitch class (pitch class 8) in a minor key. Regardless of any specific explanation for this result, it seems clear that, in broad strokes, the false alarm analyses agree with the previous findings. And most importantly, these results replicate the intriguingly novel finding of a false memory effect for musical stimuli, based on perceived expectancy for these stimuli.

Finally, *c* values for each participant in each target condition were submitted to a One-Way repeated measures ANOVA with *Target Note* as a factor. There was a significant effect of *Target Note, F*_(2, 38)_ = 5.125,*MSE* = 0.162, *p* = 0.011, *η*^2^_*p*_ = 0.222; indicating that participants had a marginally more liberal response criterion for trials with high expectancy tones than medium expectancy tones, *t*_(19)_ = 2.225, *p* = 0.038, and in turn a more liberal response criterion for medium than low expectancy tones, *t*_(19)_ = 7.738, *p* < 0.001. Figure 4C presents the means (and *SE*s) for this effect. This result is consistent with the results from the false alarms analysis, with more liberal bias leading to higher false alarms for high and medium expectancy targets.

Theoretically, these findings confirm the viability of the congruence account developed earlier, in which differential memory is shown for schema-congruent events, relative to schema-incongruent events. In contrast, this study failed to reveal any support for the distinctiveness account in musical memory, with unexpected events no longer “popping out” from their tonal background. Although not immediately obvious, such a result is understandable with reference to the fact that the minor tonality actually has multiple forms, with these multiple versions of the minor psychologically accessible to listeners at some level (see Vuvan et al., 2011, for a review and relevant data). Because the low expectancy event in this study does occur in one of the three minor forms (specifically, the ascending component of the melodic minor) it could be considered to be not as truly distinctive in a minor tonal context. As such, it would no longer pop put from its tonal background. As an aside, recognizing the more ambiguous tonal function of this tone in a minor context arising from these different forms does not undermine the distinction between the degrees of expectancy for the moderate and low expectancy tones. Although the low expectancy tone does appear in one of the three minor forms, the moderate expectancy tone occurs in all three minor tonality variants. Accordingly, this note remains, on a theoretical and psychological level, more expected than the low expectancy event.

In sum, the current experiment replicated the findings of Experiment 1 in terms of showing an impact of expectancy generation on memory for individuals in a general sense, and in demonstrating the existence of a false memory effect for musical stimuli. Extending these previous findings, this study showed that manipulating, and specifically weakening, the theoretical and psychological stability of the reference schema (i.e., musical tonality) similarly modulates the effect of expectancy generation on memory. Extending this latter finding makes an intriguing prediction: If the melodic context were to be totally devoid of tonal structure, then there should be no evidence of tonal expectancies on memory. Experiment 3 examined this final prediction.

## Experiment 3: note memory in atonal melodies

The goal of this experiment was to provide a final test of the impact of expectancy generation on musical memory. In this case, the strategy employed was a logical extension of the previous experiment in which a weakening of the tonal structure of the melodic context ultimately led to weaker effects of tonality on memory. Specifically, this experiment removed all tonal structure whatsoever from the melodic contexts. If the memory differences previously observed were truly a result of expectancy generation driven by the formation of a tonal schema, then removing tonality should have the corresponding effect of removing differential memory performance. To examine this idea, all stimulus melodies were composed to be atonal, that is, to not suggest any major or minor tonality to the listener.

### Methods

#### Participants

Twenty participants (14 females; mean age = 21.2 years, *SD* = 3.1 years) who did not participate in Experiments 1 or 2 were recruited from the University of Toronto Scarborough community using posted advertisements, and were compensated $10 for their participation. The following descriptive statistics were calculated for all 20 participants. These participants had an average of 3.7 years of formal musical training (*SD* = 4.2 years), and an average of 1.8 years of musical theory training (*SD* = 0.2 years). With respect to other musical activity, on average, participants listened to music for 10.2 h per week (*SD* = 5.3 h), and played music for 1.0 h per week (*SD* = 2.6 h). None of the participants had ever participated in a music psychology experiment before, nor did any participants report a familiarity with the music cognition research literature. Finally, none of the participants reported having absolute pitch.

#### Stimuli, apparatus, and procedure

**Figure 5:**
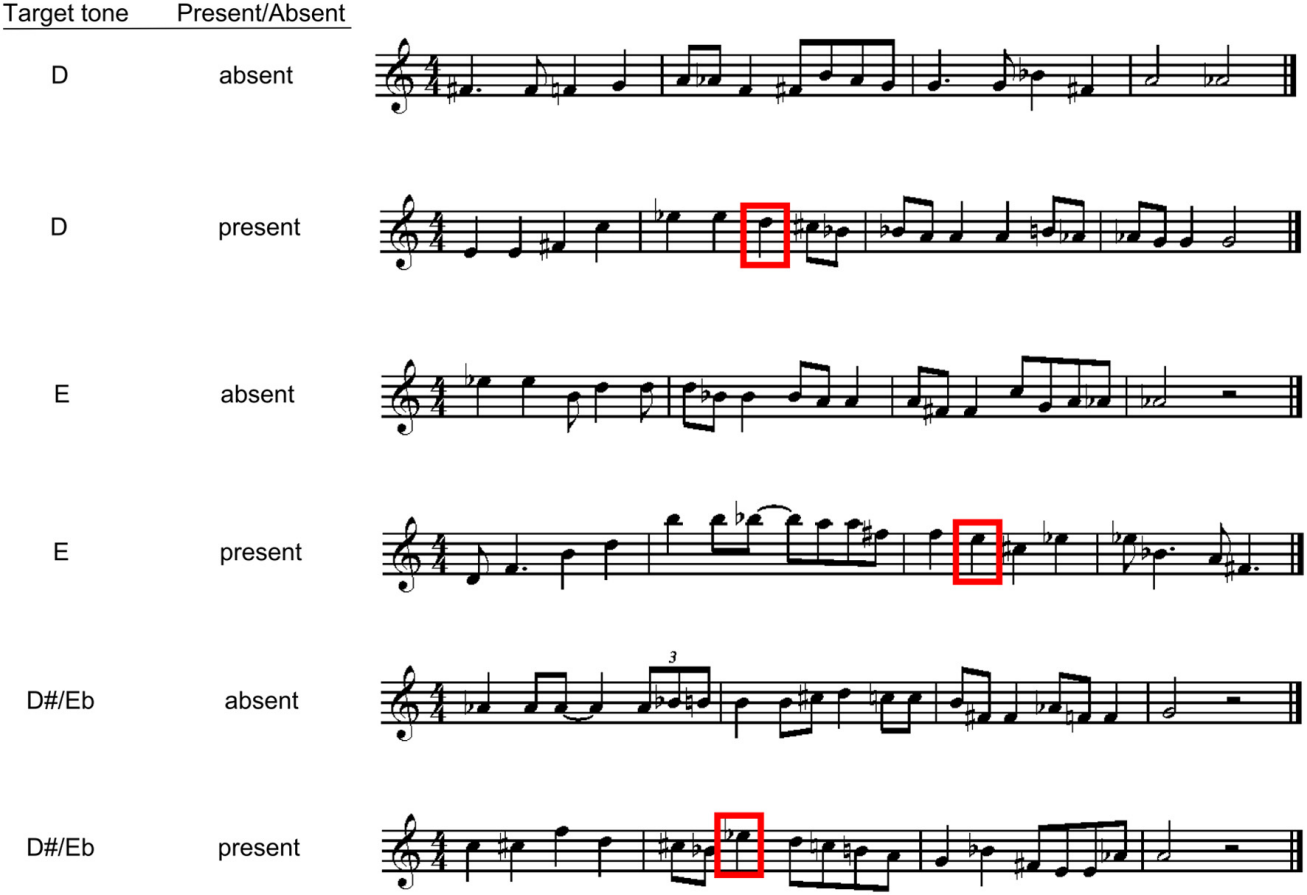
**Examples of experimental stimuli for Experiment 3**. The boxes indicate present targets.

The only difference in the current experiment from the previous two studies involved the stimulus melodies. To create the atonal stimuli for Experiment 3, the melodies from Experiment 1 were altered by modifying non-target tones by one semitone, while preserving the contour (pattern of ups and downs in pitch) of the original melody; examples of these melodies can be seen in Figure 5, with all melodies shown in Supplementary material. As in Experiments 1 and 2, the Krumhansl-Schmuckler key-finding algorithm (Krumhansl, 1990) was used to ensure that the melodies were indeed truly atonal, and did not provide any unintended tonal information. The algorithm indicated that none of the melodies correlated significantly with any particular key [mean of the maximum key correlation aggregated across all melodies *r*_(22)_ < 0.50, *SD* = 0.07, highest maximum correlation for any melody and its best fitting key *r*_(22)_ = 0.60]. Like the maximum key correlation, the second highest key correlation across melodies was relatively modest [mean *r*_(22)_ = 0.42, *SD* = 0.09]. Importantly, the maximum key correlation for this experiment was significantly lower than for Experiment 1 (correlations with G major), *t*_(142)_ = 24.020, *p* < 0.001 or Experiment 2 (correlations with G minor), *t*_(142)_ = 27.904, *p* < 0.001, indicating a much weaker sense of tonal center in these melodies.

As a subsidiary analysis, care was taken to ensure that there was no instantiation of tonality through the differential frequency of occurrence or durations of the 12 pitch classes across the melodic corpus. Previous work (Smith and Schmuckler, 2004) found that a tonal center can be instantiated simply by having one of the pitch classes occur significantly more frequently than the remaining pitch classes. Accordingly, a chi-square test on the frequency of occurrence of each pitch class, standardized for duration, confirmed that no pitch class occurred significantly more than any other one, χ^2(11)^ = 4.112, *p* = 0.967. Due to the lack of tonal schema in this experiment, all three target tones (D, E, D#/Eb) were predicted to be equally expected.

### Results and discussion

The *d*' and *c* values for each participant in each target condition were calculated in the same manner as in the previously experiments. Again, to control for the effects of musicianship, all ANOVA analyses reported for Experiment 3 were performed with musical training (in years) as a covariate. There was never a significant main effect of musical training, nor were any interactions with musical training significant, all *p* > 0.05.

**Figure 6:**
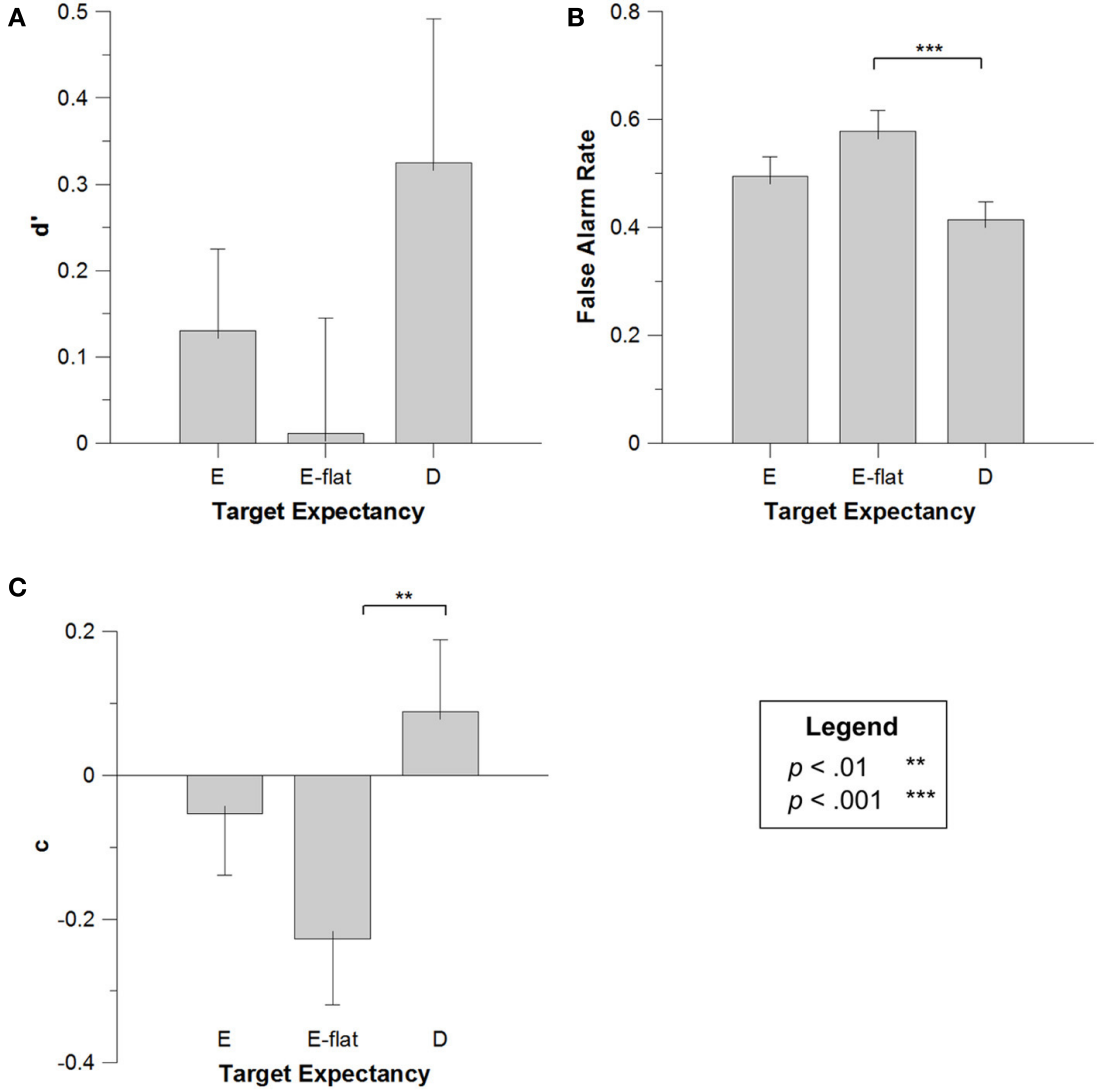
***d'* (A), false alarm rate (B) and *c* (C) for high, moderate, and low expectancy targets in Experiment 3**. Error bars depict the standard error of the mean.

In this experiment, participant performance, as measured by *d*', was not significantly better than chance (*d*' = 0), *t*_(19)_ = 1.716, *p* = 0.102. *d*' values were submitted to a One-Way repeated measures ANOVA with *Target Note* (D vs. E vs. D#/Eb) as the sole factor. This analysis failed to reveal any effect of target on *d*',F_(2, 38)_ = 0.993 *MSE* = 0.312, *p* = 0.380,*η*^2^_*p*_ = 0.052; Figure 6A presents the mean *d*' values as a function of target note. Thus, in keeping with our earlier predictions, eliminating the tonal schema from the melodies eliminated the congruency and distinctiveness effects on memory for melody tones. In other words, removing the theoretical and psychological hierarchies of stability produced melodies that failed to generate differential expectancies for tones; without any tonality-based expectancy there was no corresponding effect on note memory.

A subsequent analysis examined false alarm rates, employing a One-Way repeated measures ANOVA, again with *Target Note* as a factor. Here, the effect of target was significant, *F*_(2, 38)_ = 9.989,*MSE* = 0.011, *p* < 0.001,*η*^2^_*p*_ = 0.357; Figure 6B presents the mean false alarm rates for the different target notes. Multiple Bonferroni-corrected comparisons (critical *p* = 0.017) indicated that this effect was due to more false alarms for D#/Eb than for D, *t*_(19)_ = 6.577, *p* < 0.001; these differences can be seen in Figure 6B. The occurrence of significantly more false alarms for D#/Eb is somewhat unexpected. One possible explanation for this result would be that this tone was sounded longer than the other target tones in the context, and thus became slightly more expected based on differential duration. Unfortunately for this explanation, there was no evidence that the cumulative duration (across all of the stimulus melodies) of the D#/Eb target tone (~42 s) was any different than either the D target (~45 s) or the E target (~39 s). Alternatively, it might be that this differential performance arises through more overall differential experience with the various frequencies of occurrence of the 12 pitch classes based on a general result of musical acculturation. Some fascinating evidence over the years (Simpson and Huron, 1994; Ben-Haim et al., 2013) has found that, looking across broad corpuses of music, there are systematic differences in real-world exposure to the 12 pitch classes, and that this differential exposure influences pitch perception and memory. Unfortunately, these analyses have typically found that the tone D occurs far more commonly than the tones D#/Eb (Simpson and Huron, 1994; Ben-Haim et al., 2013). As such, this hypothesis fails to account for the current findings. Irrespective of an explicit explanation for why the D#/Eb tone showed elevated false alarm rates, the current findings are consistent with the notion that the removal of a tonal center from these melodies eliminated any systematic, explainable differences in memory for the individual target tones.

Next, *c* values for each participant in each target condition were submitted to a One-Way repeated measures ANOVA with *Target Note* as a factor. There was a marginally significant effect of *Target Note, F*_(2, 38)_ = 3.090,*MSE* = 0.128, *p* = 0.058, *η*^2^_*p*_ = 0.147, with comparisons indicating that participants had a significantly more liberal response criterion for D#/Eb target trials than D target trials, *t*_(19)_ = 3.113, *p* = 0.006. Figure 6C presents the means (and *SE*s) for this effect. This result is consistent with the results from the false alarms analysis, with more liberal bias leading to higher false alarms for D#/Eb than D targets. Finally, by way of explaining the unexpected false alarm and bias effects in this experiment, it is worth noting that performance in Experiment 3 (as measured by *d*') was significantly worse that in Experiment 1, *t*_(38)_ = 3.640, *p* = 0.001, and Experiment 2, *t*_(38)_ = 4.478, *p* = 0.00007, and that Experiment 3 was the only one of the experiments in which participants' *d*' values did not significantly different from 0. Thus, it is clear that participants struggled with the atonal melodies, supporting the idea that lack of tonal structure prevented participants from using typical memory strategies (such as availability or distinctiveness) in this experiment.

## General discussion

The current series of experiments demonstrated the influence of melodic expectancies on memory for individual musical tones. Specifically, Experiment 1 demonstrated that when a tonal schema was strongly evoked by a major melodic context, memory was enhanced for both schematically congruent and incongruent tones, with congruent tones also falsely remembered when they were not actually present in a melody. Experiments 2 and 3 extended this initial result by systematically reducing the strength of this tonal schema through the use of a minor tonality (Experiment 2) and an atonal context (Experiment 3), with this manipulation leading to a progressive weakening of the memory effects observed in the initial experiment.

Theoretically, these findings support the operation of two different processes affecting memory for two categories of tones within these melodic contexts. First there is evidence for a congruency account, in which events that are schematically consistent with the overall context, in this case the overarching musical tonality, are better remembered than events that are less consistent with this context. Evidence for the congruency account appeared in both Experiments 1 (in terms of *d*' and FA/bias) and 2 (in terms of FA/bias only). Second, there was also evidence for the distinctiveness account, as demonstrated by better memory for schematically inconsistent tones within the overall context; this evidence was only seen in Experiment 1.

On its own, the demonstration of a distinctiveness effect in musical memory as a function of expectancy generation is a noteworthy result. Previous research by Schmuckler (1997) on the relation between expectancy and memory failed to find any support for a distinctiveness account in memory for melodies. With regard to this earlier work, however, it is important to remember that Schmuckler manipulated expectancy levels of melodies by randomizing the occurrence of the pitch events within the final two measures of a set of eight measure melodies, while holding the rhythmic structure of these final two measures constant. Accordingly, these variations did not introduce tones that were strongly schematically inconsistent with the overall tonality of the melodies. Thus, in this earlier project expectancy manipulations occurred via varying the co-occurrence of the pitch events with their corresponding metrical positions within these melodies (see Prince and Schmuckler, 2014, for a discussion of tonal-metric correlations), thereby likely resulting in alternatives that did not present as wide a range of perceived expectedness as the current paradigm. Put more simply, in this earlier study there were likely no melodies that were as “unexpected” as the unexpected tones in the current study. Given this possibility, it is not at all surprising that this previous project did not produce evidence for a distinctiveness account, as none of the melodies were as “distinctive” as the unexpected targets were in this series of experiments. It would be interesting to repeat that previous work, examining memory for entire melodies that systematically vary outside of a tonal framework, to see if both congruency and distinctiveness accounts can be similarly simultaneously operative in melodic memory.

On a parallel theoretical level, and as discussed previously, both the congruency and distinctiveness accounts can be aligned with the operation of different types of memory heuristics. Specifically, the congruency effects can be considered in relation to Tversky and Kahneman's (1973) classic work on the availability heuristic, which would predict that schema congruent tones, because they are more present and hence available, would be better remembered when they occurred, and more falsely remembered when they did not occur; both of these predictions were borne out in our results. In contrast, distinctiveness effects can be considered in relation to the distinctiveness heuristic (Israel and Schacter, 1997; Schacter et al., 1999, 2001; Dodson and Schacter, 2002; Schacter and Wiseman, 2006), which would predict better memory for tones that are schematically unexpected, a prediction that was also confirmed by our results. To our knowledge, the current findings are the first to explicitly place, and demonstrate, the operation of such memory heuristics in musical memory.

More important than simply identifying the occurrence of these heuristics in a musical context, however, is the evidence for the simultaneous operation of these two processes. As discussed by Schacter and Wiseman (2006), very little work has examined how the different memory heuristics function in relation to one another. In this sense, it is worth highlighting that, at least within the current context, both heuristics simultaneously influenced memory performance, and did so without interfering with one another. Accordingly, our data suggest that these two processes can operate in a complementary fashion in the proper circumstances. In fact, the potential for a complementary relation between these two processes has been noted by others. Tversky and Kahneman (1973) observed that when an item possesses distinct features, it is better encoded in memory, which contributes to its mental availability. In other words, both distinctiveness and availability may be seen as simply two paths by which in a schematic framework can affect memory processes.

One assumption cutting across all of the experiments outlined here is that the schematic frameworks (i.e., tonal structures) engendered by the melodic contexts produced expectancies for upcoming events that subsequently influenced memory performance. Moreover, and somewhat critical within this framework, is the idea that variation in the schematic frameworks (i.e., major vs. minor vs. atonal) also produced expectancies for upcoming events that varied in their subsequent strength and degree of specificity. Thus, major contexts produced the strongest and most specific expectancies, followed by minor contexts, and finally by atonal contexts. Interestingly, although there is evidence that different tonal contexts produce different forms of schematic frameworks that vary in their degree of psychological stability (Krumhansl et al., 1982; Harris, 1985; Delzell et al., 1999; Vuvan and Schmuckler, 2011), and that expectancies do indeed vary in their strength and specificity (Schmuckler, 1989, 1990), there is actually no data explicitly linking different tonal frameworks to varying forms of musical expectancies. In this regard it is worth nothing that virtually all of the work on expectancy employing Western tonal melodic contexts (e.g., Schmuckler, 1989, 1990, 1997; Schellenberg, 1996, 1997; Larson, 2002, 2004; Margulis, 2005, 2007; Pearce and Wiggins, 2006, 2012; Thorpe et al., 2011) have either employed, or at least been applied to, major tonal contexts. Similarly, research examining harmonic priming (e.g., Bharucha and Stoeckig, 1986, 1987; Tekman and Bharucha, 1998; Tillmann et al., 1998, 2000, 2003; Bigand et al., 2003; Marmel et al., 2008, 2010; Marmel and Tillmann, 2009) has almost exclusively employed major tonality chord progressions. Accordingly, the impact of minor tonal frameworks on musical expectancy formation has been virtually unstudied; as such, it is unclear as to whether minor tonalities actually give rise to expectancies that are less strong and/or specific.

Finally, two caveats to our findings are in order. First, one of the central debates in the literature on musical expectancy has to do with whether the locus of expectancy effects is sensory (e.g., repetition of pitches, spectral characteristics of the target) or cognitive (e.g., based on mental representations of the tonal hierarchy), as we have argued here. In the current study, we did not explicitly control sensory factors in our stimuli, thus leading to the possibility that our expectancy results could be explained by sensory factors rather than the cognitive factors we have invoked here. In a recent review, Collins et al. (2014) analyzed the stimuli from seven different tonal priming experiments, and showed that participant reaction times could be modeled by information from periodicity pitch (sensory), chroma vectors (cognitive), and activations on tonal space (cognitive). Collins et al. reported a significant contribution of cognitive factors for all experimental data tested. Moreover, these authors found that tonal space variables (i.e., tonal hierarchy representations) explained more variability in reaction times than did periodicity pitch variables, which suggests a greater role for cognitive than sensory factors in musical expectancy. Due to lack of stimulus control, we cannot conclude with certainty that our results emanate from cognitive rather than sensory sources. However, the results of Collins et al. (2014), as well as evidence from studies indicating that listeners do store tonal representations in long-term memory (Bigand et al., 2003; Marmel et al., 2010; Vuvan and Schmuckler, 2011) argue against the idea that short-term sensory memory alone can account for listener behavior in studies of tonal perception (as suggested by Leman, 2000). Rather, these previous findings suggest that our results are likely to emanate at least in part from cognitive expectancy.

As a second caveat, one might wonder whether the schematic expectancies generated by these stimuli effect memory for tones as a result of encoding, retention, or retrieval aspects of memory processing. On the one hand, the distinctiveness heuristic has been traditionally discussed as an encoding effect. On the other hand, the availability heuristic mechanism is inherently retrieval-oriented, given that it is items whose exemplars are more mentally available during retrieval that are better remembered. It should be noted, though, that this orientation toward retrieval does not preclude effects during encoding and retention, such as distinctiveness, from having an impact upon availability at retrieval. Ultimately, the elucidation of the locus of memory effects is a historically difficult issue, and unfortunately the current set of experiments cannot adjudicate between these varying accounts. Ongoing work is currently focusing on adapting this memory paradigm in an attempt to disentangle these different accounts. One straightforward means of at least partially addressing this concern is to present the probe tone before the melody containing the target, thus (presumably) ensuring that the tone of interest will be well-encoded. If the expectancy effects observed here persist in such an experiment, this would provide evidence that this effect is not dependent on differential encoding. An additional benefit of such an experiment would be to assess whether availability-based memory effects are operating at encoding, or retention or retrieval.

In summary, the three experiments presented here provide evidence for tonal schematic effects on memory for tones in a melody. Although illuminating, these findings have only begun to explore the complex relation between musical schema formation, expectancy generation, and subsequent memory for musical materials. Ultimately, the hope is that such work will deepen our understanding of how the mind processes and retains not only musical information, but complex auditory information more generally.

### Conflict of interest statement

The authors declare that the research was conducted in the absence of any commercial or financial relationships that could be construed as a potential conflict of interest.

## Footnotes

^1^A subsidiary analysis in this experiment, as well as the forthcoming studies, examined hit rate as a function of serial position with the melody, coded as position 1 (measure 2, beat 2), 2 (measure 2, beat 3), 3 (measure 3, beat 2), 4 (measure 3, beat 3). Across all three of the studies these analyses demonstrated significant differences, or marginally so, as a function of serial position, with the best memory for serial position 4, followed by serial position 3, and so on. Thus, in keeping with a standard recency account (Ebbinghaus, 1913; Feigenbaum and Simon, 1962; Murdock, 1962), all of these studies showed better memory for the most recently presented information.
